# Evaluating the efficacy of Mazao Tickoff (*Metarhizium anisopliae* ICIPE 7) in controlling natural tick infestations on cattle in coastal Kenya: Study protocol for a randomized controlled trial

**DOI:** 10.1371/journal.pone.0272865

**Published:** 2022-08-16

**Authors:** Joseph Wang’ang’a Oundo, Daniel Masiga, Michael Nyang’anga Okal, Gebbiena M. Bron, Komivi S. Akutse, Sevgan Subramanian, Quirine ten Bosch, Constantianus J. M. Koenraadt, Shewit Kalayou

**Affiliations:** 1 International Centre of Insect Physiology and Ecology (ICIPE), Nairobi, Kenya; 2 Quantitative Veterinary Epidemiology, Wageningen University & Research, Wageningen, The Netherlands; 3 Laboratory of Entomology, Wageningen University & Research, Wageningen, The Netherlands; Federal University of Western Pará, BRAZIL

## Abstract

Ticks and tick-borne diseases cause substantial economic losses to the livestock industry in sub-Saharan Africa. Mazao Tickoff is a novel bioacaricide developed for tick control and is based on the entomopathogenic fungus *Metarhizium anisopliae* sensu lato (s.l.) isolate ICIPE 7. To date, no randomized controlled study has been undertaken to demonstrate the efficacy of this bioacaricide in reducing natural tick infestation on cattle. To this end, this field trial is designed to evaluate the anti-tick efficacy of Mazao Tickoff on cattle in coastal Kenya compared to a standard chemical tick control protocol. In this prospective, multi-center randomized controlled trial, eligible herds will be randomized by the herd size to the intervention arm in a 1:1:1 ratio to either Triatix^®^ (active ingredient: amitraz); Mazao Tickoff (active ingredient: *M*. *anisopliae* ICIPE 7); or placebo (excipients of the Mazao Tickoff), with a total enrollment target of 1,077 cattle. Treatments will be dispensed on Day 0 (defined individually as the day each animal receives the first treatment) and thereafter every two weeks until Day 182. Ticks will be counted on every animal in each herd (herds to be included have at least one animal bearing at least one tick on Day 0), and thereafter on bi-weekly intervals until Day 182. The primary efficacy assessments of Mazao Tickoff will be based on the mean percentage reduction in tick counts at each post-treatment follow-up visit compared to the placebo group and the Triatix^®^ arm. Further, the effect of Mazao Tickoff on the prevalence of common cattle pathogens, *Anaplasma marginale* and *Theileria parva*, will be determined by assessing incidence and seroprevalence at four different time points. This protocol describes the first rigorous evaluation of the efficacy of Mazao Tickoff and its potential as a viable alternative non-chemical acaricide tool for tick control in Kenya and elsewhere.

## 1. Introduction

Ticks (Acari: Ixodida) are obligate blood-feeders that parasitize mammals, birds and reptiles. They transmit various pathogens, including protozoa, bacteria, and viruses, that cause a significant burden to human and animal health [1, 2]. The severity of the disease depends on the pathogen involved and the immunity of the infected host. The most economically important ixodid ticks in Kenya and sub-Saharan Africa belong to the genera of *Amblyomma*, *Hyalomma* and *Rhipicephalus* including the sub-genus *Boophilus* [3]. The most widespread TBDs of cattle in Kenya include anaplasmosis caused by the bacterium *Anaplasma marginale*, babesiosis caused by the protozoan *Babesia bigemina*, East Coast fever (ECF) caused by the protozoan *Theileria parva* and benign theileriosis caused by the protozoan *Theileria mutans* [4–9]. Direct losses due to heavy tick infestation on cattle include damage to hides and skins, damage to teats and reduced productivity, decrease in live-weight gain, blood loss, suppression of immunity, cause nuisance and skin irritation and introduction of toxins [3, 10, 11]. This highlights the importance of controlling tick infestations in cattle.

Chemical acaricides are the major component of tick prevention and control programs in livestock [12]. However, the emergence and spread of acaricide resistance threaten the long-term sustainability of this method [13, 14]. Furthermore, heavy reliance on these acaricides has also resulted in contamination of milk and meat products with chemical residues in addition to environmental contaminations [15]. This necessitates the development of new sustainable alternatives, including the use of biological control agents.

Entomopathogenic fungi such as *Metarhizium anisopliae* have shown great potential as biocontrol agents of ticks [16–18]. This fungus is virulent against all stages (egg, larva, nymph and adult) of *Amblyomma variegatum*, *Rhipicephalus appendiculatus*, *Rh*. *evertsi* and *Rh*. *decoloratus* ticks when these come into contact with the conidia of the fungus [16, 18–21]. The fungus also reduces engorgement, egg production and hatching rate, and larval and nymphal molting rate in the surviving population of treated ticks [18, 22–24]. It was recently shown in limited on-farm trials that *Metarhizium anisopliae* sensu lato (s.l.) isolate ICIPE 7is pathogenic to both amitraz-resistant and susceptible *Rh*. *decoloratus* [25]. This fungus is compatible with other chemical acaricides and some semiochemicals/attractants [25–30]. A rigorous randomized control field efficacy trial with *M*. *anisopliae* s.l. ICIPE 7 against ticks needs to be undertaken in natural field conditions.

Mazao Tickoff is a near-commercial bioacaricide developed as a possible tool for resistance management in ticks and an alternative to chemical acaricides (https://realipm.com/products/mazao-tickoff/). This bioacaricide provides a broad spectrum of acaricidal activity by combining the acaricidal properties of *M*. *anisopliae* ICIPE 7 with the repellent properties of kerosene. To date, existing data supporting the efficacy of Mazao Tickoff are limited to small-scale pilot trials only (https://patents.google.com/patent/WO2017216752A1/en). There is, therefore, a need to establish proof of efficacy in the natural field conditions before its adoption and upscaling can be recommended. To this end, this study protocol describes the methodological approach for evaluating the efficacy of Mazao Tickoff against natural tick infestation on cattle in coastal Kenya.

## 2. Trial objectives

### 2.1 General objective

To determine the efficacy of the fungal-based product Mazao Tickoff against natural tick infestation and tick-borne diseases on cattle and evaluate its potential as an alternative to the standard chemical acaricide, amitraz in small-holder farming systems in coastal Kenya.

### 2.2 Specific objectives

1. To assess the efficacy of Mazao Tickoff in reducing tick infestation level on cattle, compared to the placebo.
2. To assess the efficacy of Mazao Tickoff in controlling natural tick infestation on cattle compared to the chemical Triatix^®^ acaricide in a non-inferiority test.
3. To determine the impact of Mazao Tickoff on the prevalence and transmission dynamics of *A*. *marginale* and *T*. *parva* on cattle measured by seroconversion, with subsequent high-resolution melting PCR (HRM-PCR) used to estimate the prevalence of active infection.
4. To assess and document any adverse events that may occur among cattle receiving Mazao Tickoff.

## 3. Materials and methods

### 3.1 Study setting

**Fig 1:**
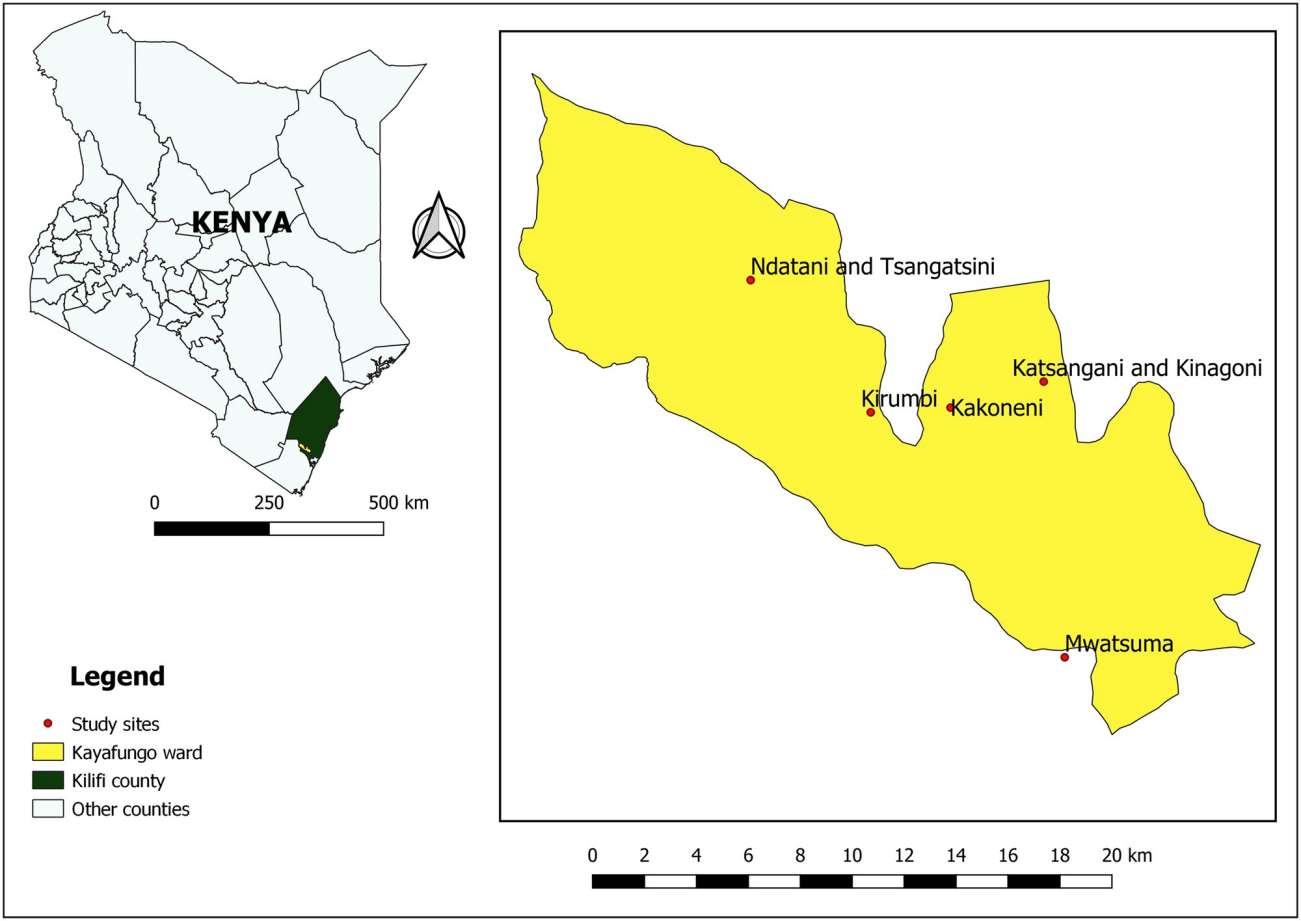
Map of Kayafungo ward in Kilifi county in coastal Kenya showing the trial sites. This map is republished with data under CC BY licenses from the following sources: https://africaopendata.org/dataset/kenya-counties-shapefile from openAfrica, 2015 [36]; and https://gadm.org/download_country_v3.html from GADM, 2018 [37].

The study will be conducted in Kayafungo ward, Kaloleni sub-county in Kilifi County in coastal Kenya (Fig 1). The region is hot throughout the year with an average temperature range of 23°C—34°C. The altitude range is between 0 and 464 m above sea level. The region experiences two rainy seasons, i.e., from April to June and October to November, but some rain falls nearly every month, especially near the coastline. The total precipitation varies from 900 to 1500 mm per annum along the coastal belt to 500–600 mm in the backcountry [31–33]. The study area is suitable for the current trial as tick infestation is common in the selected sites. Further, previous reports have demonstrated that tick-borne infections are prevalent in the coastal region [5, 7, 34, 35].

### 3.2 Cattle herds, management, and tick infestation exposures

Cattle herds in the study area are typically small-holder herds (average herd size of seven cattle per herd) of the indigenous zebu (*Bos indicus*) breed. Most of these cattle are maintained under traditional extensive management systems. During the day, adult and weaner cattle of several herds graze together in a common pasture on fallow or communal grazing land. These animals also obtain drinking water in the same water pans that are scattered in the villages. Therefore, adults and weaner zebu of a village are likely exposed to a similar tick habitat with similar tick infestation levels. Young calves are usually tethered around the homestead or grazed separately from the dams until they are weaned. Before this trial, a cross-sectional survey was conducted during the dry season in 14 villages to assess the species diversity of ixodid ticks infesting cattle and their burden and prevalence of associated pathogens. This unpublished survey recorded a 25% (range 22–29.1%) tick infestation prevalence. All the examined herds (n = 95) had at least one animal infested by a tick. The median infestation intensity was four ticks per animal (Range 1–19). The corresponding figure at the herd level was 12.7 ticks (range: 8.7–19.4). As expected, the prevalence and intensity varied across the villages. For this trial, we identified four village clusters 3.5–5 km2 size with distinct animal grazing patterns and intensities.

### 3.3 Trial design

**Fig 2:**
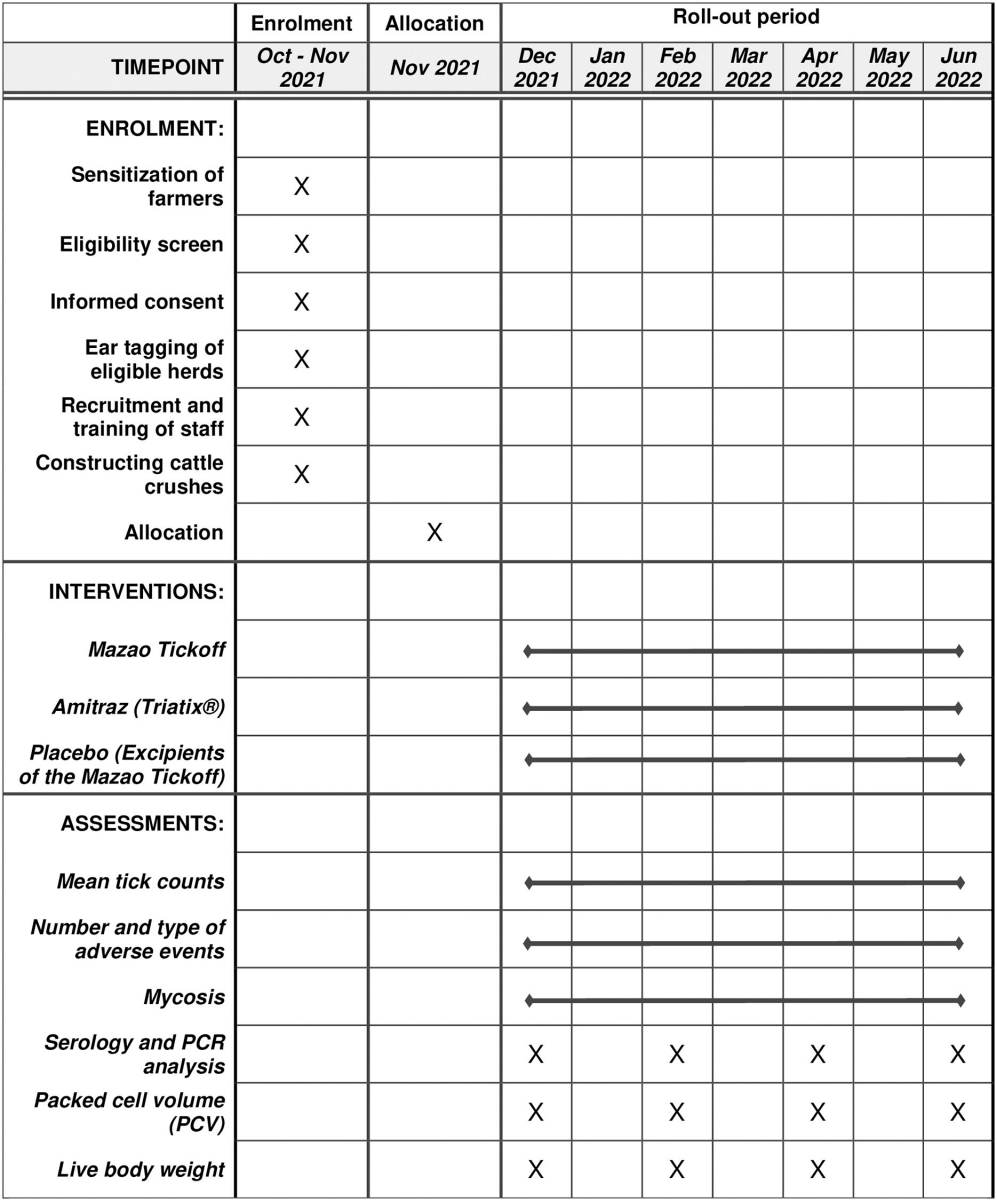
Schedule of enrolment, interventions, and assessments.

This study is a prospective, multi-center randomized controlled field trial designed to evaluate the efficacy of Mazao Tickoff. The study will be conducted during the dry and rainy season, from December 2021 to June 2022. The rainy period, and soon thereafter, is characterized by a high abundance of *Rh*. *appendiculatus*, *Rh*. *decoloratus* and *Am*. *variegatum* [3, 38–41] and favorable for high fungal growth [18]. The schedule of trial activities is presented in Fig 2. Local zebu cattle managed under an extensive grazing system will be enrolled in the study based on evidence of infestation with live attached ticks, generally in good health, and the owners’ willingness to participate in the study. Zebu cattle are the predominant livestock species in the area and were therefore chosen for this study. Qualifying herds will be stratified by size and the study village clusters, and randomly allocated to receive either Triatix^®^ (Cooper K-Brands Ltd); Mazao Tickoff; or placebo in a 1:1:1 ratio. If after randomization we note a large variability in tick counts among the intervention arms, then we will include tick infestation prevalence and intensity as part of the randomization plan. To reduce the likelihood of spillover effect with biopesticides, all cattle from the same herd will be treated using the same product and will remain with their owners under their natural grazing conditions throughout the trial. For logistical convenience, we will construct three crushes in each village cluster. Cattle will receive treatment on Day 0, and thereafter every two weeks until Day 182. Day 0 will be defined individually as the day an animal receives the first treatment. Whole-body tick counts will also be done on Day 0, and thereafter at two-week intervals until Day 182. To ensure chemical acaricide is not used after the experimental treatments, farmers will be incentivized for adhering to our trial protocol. This will be in the form of providing cattle with strategic deworming (twice during the project period) and free veterinary consultation and treatment in case any cattle show symptoms of trypanosomosis or tick-borne diseases. Study completion will be the day the animal will complete the study, normally on Day 182 unless it is prematurely withdrawn from the study. Individual animals will be the experimental units for statistical analysis of tick infestations.

### 3.4 Sample size determination

This trial proposes to assess the efficacy of Mazao Tickoff in reducing on-host tick counts compared to a commonly used chemical acaricide. Our previous small-scale field trial showed that, two weeks after the last application of Mazao Tickoff, the average number of ticks on the treated cattle (47.2 ± 8.8) was significantly lower than the untreated cattle (338.7 ± 12.8), representing 86.1% protection (https://patents.google.com/patent/WO2017216752A1/en). Similarly, a weekly application of a Triatix^®^ acaricide for four weeks resulted in a 94.9% reduction of tick infestation counts on cattle [25]. Based on a significance level of 5%, and assuming a 95% efficacy in the reference Triatix^®^ acaricide arm [25], a minimum sample size of 138 zebu cattle per intervention arm will provide 80% power to detect a difference in efficacy if the Mazao Tickoff bioacaricide is no more than 10% inferior to the reference arm. A non-inferiority margin of 10% below a Triatix^®^ acaricide efficacy of 95% is considered acceptable given the limited availability of possible alternatives for tick control and the added advantages of *M*. *anisopliae* in biological control of ticks, i.e., selective and virulent against all tick stages [16–22, 42], pathogenic to both acaricide-resistant ticks [25], and is safe to humans, animals and the environment [43].

The sample size was calculated using a previously described formula [44]:

$$n=[{\left(Z_{\alpha /2\;}+\;Z_{\beta }\right)}^{2}\;\times \;\{(p1\;(1-p1)\;+\;(p2\;(1-p2))\}]\;/\;{\left(p1\;-\;p2\right)}^{2}$$

where:

n = sample size required in each intervention arm,

p1 = protection efficacy of Triatix^®^ = 0.95,

p2 = protection efficacy of Mazao Tickoff = 0.85,

p1—p2 = minimum worthwhile difference = 0.10,

Z_α/2_: for 5% level of significance = 1.96,

Z_β_: for 80% power = 0.84.

Herd-level treatments and repeated measurements are likely to enhance intra-herd clustering of measurements estimated at individual animal levels. To account for the variation that may occur among herds, i.e., clustering effect, inflating the sample size by two- to four folds can account for the potentially large variation among clusters [45]. We, therefore, inflated the sample size by two folds and obtained a total of 276 zebu cattle per intervention arm. A dropout rate of 30% was included in the calculation to account for potential dropouts during the trial, bringing the total number of cattle per study to 359. The total sample size for all the three treatments together being 1,077 zebu cattle.

### 3.5 Eligibility criteria

Enrolment in the study will be restricted to cattle herds fulfilling the following inclusion criteria: (i) all cattle in the herd are of local zebu breed managed under an extensive grazing system; (ii) at least one cattle in the herd is infested with live attached hard tick(s); (iii) all cattle in the herd are apparently healthy or have minor ailments judged not to interfere with the study; (iv) there is an informed consent given by the owner or by an authorized representative. A herd will be ineligible if the individual animals are severely ill, require intensive veterinary care before enrolment, or have pre-existing medical conditions judged to interfere with the study. The eligible herds will be enrolled in the study, and the following baseline characteristics recorded: age, sex, and live body weight. Each animal will receive an ear tag containing a number of three-digit identification (ID) (village acronym/ herd number /individual number). All the animals will remain with their owners throughout the study and will be fed their usual diet with access to water according to their normal routine.

### 3.6 Allocation and randomization

The qualifying herds will be stratified by herd size and study village cluster and randomly allocated to either Triatix^®^ (Cooper K-Brands Ltd), Mazao Tickoff (Real IPM Kenya Ltd), or placebo in a 1:1:1 ratio. If, after randomization, we note a large variability in tick infestation prevalence and intensity among the groups, tick intensity and prevalence will then be considered as part of the randomization plan. All cattle from the same herd will be randomized to the same treatment. The stock solution of Mazao Tickoff (4.05 × 10^10^ conidia/mL), Triatix^®^ (Amitraz, 12.5% EC) and the placebo (excipients of Mazao Tickoff) will first be diluted at a dosage of 2 ml/L as recommended by the manufacturer before being applied on the cattle (4 liters per animal). The formulation of Mazao Tickoff will be prepared five to ten days right before application.

### 3.7 Treatment

Three treatments are deployed: Triatix^®^, Mazao Tickoff and Placebo. Triatix^®^ contains 125 g of Amitraz. Mazao Tickoff is a formulation of *M*. *anisopliae* ICIPE 7 (4.05 × 10^10^ conidia/mL) prepared in canola oil (95%) and mixed with 0.05% Triton X-100 (1.5%) and Kerosene (3.5%). The placebo contains only the excipients of Mazao Tickoff.

**Fig 3:**
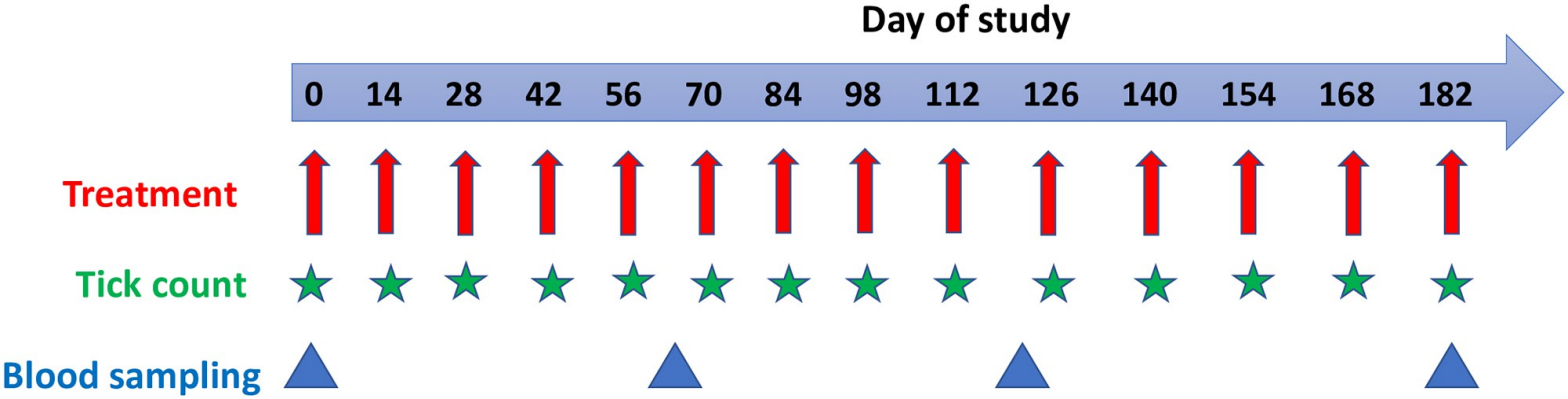
Design of the treatment regime. Administration of treatments (red arrows), time-points of tick counting (green stars) and time-points for serum and whole blood sampling (blue triangles).

Treatments will be administered topically every two weeks from Day 0 to Day 182 (Fig 3), using a hand rocker sprayer with a cone-type nozzle and a pressure of 6 kg/cm. Day 0 will be defined individually as the day an animal receives the first treatment. The animals will be restrained in a crush and then sprayed from the bottom up and in the opposite direction to how the hair lies, giving greater attention to the areas most affected by ticks, such as the inner thighs, dewlap, tail, belly, inside ears, legs, and perineum. Animals will be treated in the morning (6–8 a.m.) to avoid the adverse effects of sunlight (UV radiation) on the fungus [46]. For logistical convenience, we will construct three crushes in each village cluster and all treatments will be administered (per herd) in each crush.

### 3.8 Tick counts

**Fig 4:**
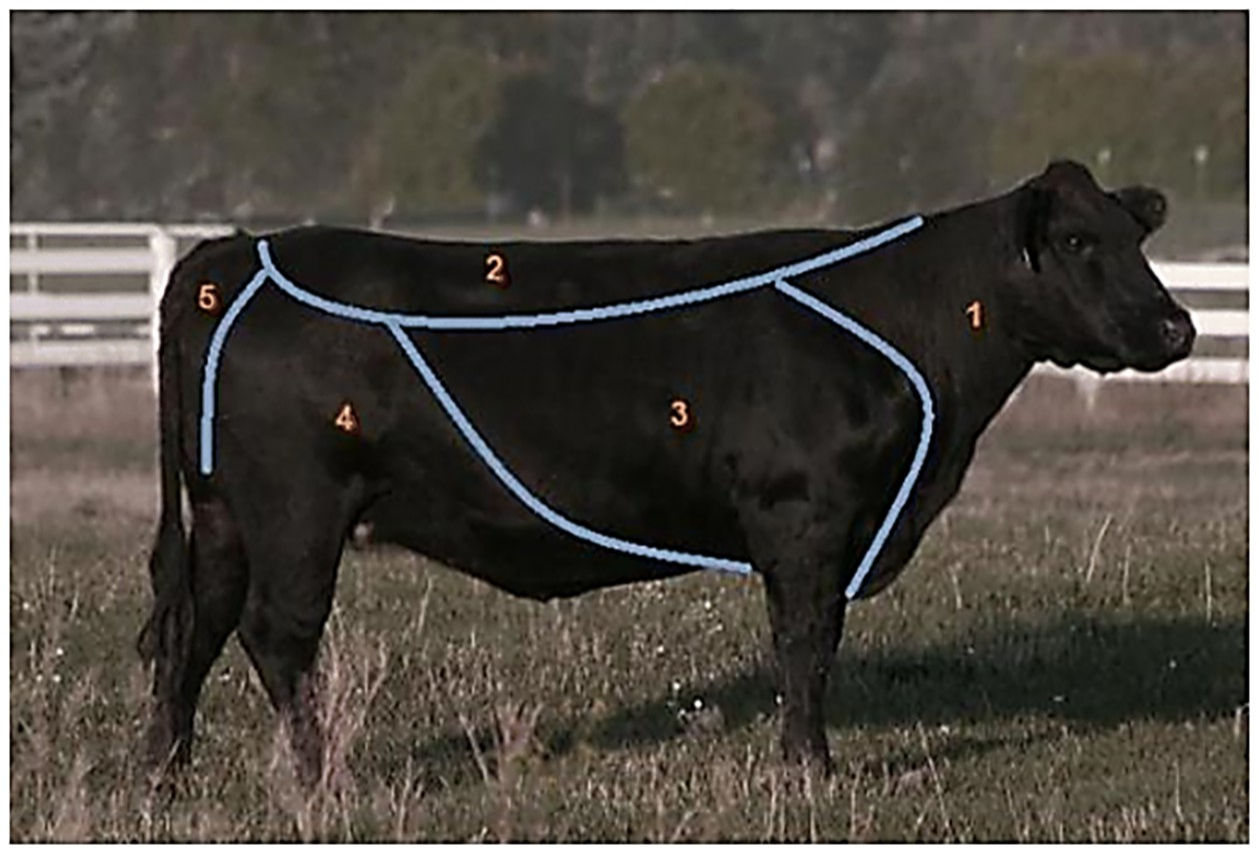
Anatomical zones (Z) differentiated for tick counting. Z1: head, ears, neck and dewlap to the point of the sternum; Z2: back and loin; Z3: forelegs, shoulders and ribs; Z4: rear legs, udder/scrotum, fore and rear flank; Z5: rump and tail. Image adapted from [47].

The cattle will be restrained and whole-body tick counts will be done on each cattle at pre-treatment on Day 0, and thereafter on bi-weekly intervals until Day 182 (Fig 3). The on-host ticks will be counted just before each spraying. Further, we will determine the feeding status of the ticks *in situ* on all the experimental animals. The animals will be restrained in a crush and carefully examined for the presence of live attached ticks in five anatomical zones (Fig 4) as described by Rocha et al. [47]. The counting will be done sequentially from zone 1 to 5 while the ticks are still attached to the animal body.

### 3.9 Fungal activity and viability test

To check on fungal activity and possible spillover effects, representative ten ticks of each species, life stage and feeding status will be collected (with forceps so as not to damage the mouthparts) from randomly selected treated animals from each intervention arm every two weeks. They will be placed in a sterile Petri dish (at most 10 ticks per dish) and maintained at a temperature of 26 ± 1°C and 85 ± 5% relative humidity (RH) for 2 weeks. Mortality will be recorded daily and dead ticks will be immediately removed and transferred to another sterile Petri dish lined with moistened filter paper to allow fungal growth on the cadaver [25].

For purposes of this experiment, we will use an already formulated product i.e., Mazao Tickoff and not the fungus as a stand-alone ingredient. However, the viability of conidia will be determined before the formulation of Mazao Tickoff by spray-platting 0.1 ml of the conidial suspension (titrated to 3 × 10^6^ conidia/mL) on Sabouraud Dextrose Agar (SDA) plates. Plates will be incubated at 26 ± 2°C for 18 hours. Sterile microscope cover slip will then be placed on each plate and the percentage of germination will be determined by counting 100 spores for each plates, using a compound microscope at 40× magnification. Germination rates > 80% after 24 hours on SDA will be considered adequate for use in the field trials. To guarantee that the product will reach its highest efficacy in the field, Mazao Tickoff formulation will be freshly prepared and used within five to ten days. The product will also be stored and transported at room temperature.

### 3.10 Estimation of tick-borne pathogen prevalence

A cross-sectional survey will be done on all herds participating in the trial. Approximately 10 ml of blood will be collected from the cattle by jugular venipuncture using 20cc syringes and sterile 18G needles. Four milliliters of whole blood will be transferred to vacutainer tubes coated with ethylenediaminetetraacetic acid (EDTA) and another 4 ml transferred into plain tubes coated with serum clot activator. The samples will be transported in cool boxes to the laboratory for further processing and analysis.

The serum will be processed and the antibodies against *T*. *parva* and *A*. *marginale* will be detected by enzyme-linked immunosorbent assay (ELISA) using standard methodology [48, 49]. For accurate interpretation of serological data, calves below 21 weeks old will not be sampled. This is because maternal *T*. *parva* antibodies can be detected in calves until 21 weeks of age [47] and may compromise the accuracy of the analysis.

Genomic DNA will be extracted from the EDTA whole blood samples using the DNeasy Blood & Tissue kit following the manufacturer’s protocol (Qiagen, Hilden, Germany) and then screened for the presence of *T*. *parva* and *A*. *marginale* by HRM-PCR [50] using primers described by Georges et al. [51] and De La Fuente et al. [52], respectively.

### 3.11 Blinding

Owing to the nature of the interventions, it will be impossible to blind the livestock owners to their intervention arm randomization. However, the identity of the intervention arm will be concealed from the owners until the completion of enrolment and randomization to eliminate participation bias. To maintain appropriate blinding, non-blinded trained personnel will be responsible for administering treatments to cattle and not participating in outcome assessment. Outcome assessors will neither have access to the database nor a preview of the previous tick count as the outcome will be recorded and transmitted electronically. The blinding of the statistician will be done using intervention arm identification codes.

### 3.12 Outcome measures

#### 3.12.1 Primary outcome measures

The primary outcome measure is the average number of ticks per individual cattle at each post-treatment follow-up visit in each intervention arm.

#### 3.12.2 Secondary outcome measures

1. The proportion of tick-free cattle in each intervention arm at each post-treatment follow-up visit.
2. Percentage reduction in tick numbers in treated cohorts as compared to placebo arm at each post-treatment follow-up visit.
3. The prevalence of *T*. *parva* and *A*. *marginale* in cattle in each intervention arm.
4. The number, type and severity of adverse events in cattle in each intervention arm.

### 3.13 Data collection, management, and analysis

#### 3.13.1 Data collection methods

The personnel responsible for counting ticks and administering the study products will receive training on the study protocol. Additional training sessions on tick feeding state (fully engorged, partially engorged and unfed) and live state (live or dead) will be provided to personnel performing tick counts. Moreover, they will be trained on differentiating the cattle breeds (indigenous zebu, exotic, and cross-breeds), age groups (calves, yearlings, and adults), sex (male and female) and how to measure the live-weight of the cattle. All consent forms will be initially prepared in English and translated into Kiswahili. To promote participant retention and minimize any loss to follow-up, contact details of the farmers will be maintained in a database for ease of follow-up. General climate data (rainfall, temperature and relative humidity) for the study area covering the experimental period will be obtained from Kenya’s Department of Meteorology.

#### 3.13.2 Data management

The study data will be collected and managed using Research Electronic Data Capture (REDCap) tools hosted at the International Centre of Insect Physiology and Ecology (ICIPE) [53, 54]. To ensure the confidentiality of the collected data, the database will be password protected and only accessed by the investigators. All consent forms containing names and contacts of livestock owners will be kept in a locked cabinet and the key kept by the principal investigator. Authorized representatives from the funding agency or regulatory bodies may inspect all documents and records of the trial.

#### 3.13.3 Data analysis

All data will be summarized and analyzed using R statistical software version 3.6.1 (http://cran.r-project.org/). Baseline demographic variables (age, sex, and body-weight) will be tabulated. All efficacy analyses will be conducted on both per-protocol (PP) population, consisting of all cattle without major protocol violations, and the intent-to-treat (ITT) population consisting of all cattle randomized to an intervention arm. Efficacy will be calculated using geometric and arithmetic means at each post-treatment day as the percentage reduction in live tick-counts in treated animals compared to the untreated controls using the following formula [55]:

$$Percentage\;efficacy=100-\left(\frac{T}{U}\times 100\right)$$

Where

$$T=\frac{post-treatment\;mean}{pre-treatment\;mean}in\;treated\;animals$$

and

$$U=\frac{post-treatment\;mean}{pre-treatment\;mean}\;in\;control\;animals$$

Generalized linear mixed models (GLMM) for negative binomial data using the logarithmic link function (log-linear modeling or regression, or Poisson regression) will be used to assess the effect of the treatments at the different follow-up measurements. The model will include the treatment variable (A, B, C), time-point and the interaction between the treatment variable and time-point [56] as fixed effects [option to supplement with age and sex]. We will account for village and herds effects by including herd nested within village as a random effect. Standard errors for the means will be calculated, and 95% confidence intervals will be constructed by time-point. Testing will be two-sided at the significance level of α = 0.05.

The non-inferiority of the Mazao Tickoff will be evaluated to the commercial Triatix^®^ acaricide at each post-treatment time-point using a margin (δ) of 10% at the one-sided α of 0.025 significance level. If the lower 97.5% confidence limit of the Mazao Tickoff is within the margin of non-inferiority, it will indicate that it is not less effective (non-inferior) to Triatix^®^ at that time-point. If the lower confidence limit is above the margin of non-inferiority, it will indicate that Mazao Tickoff is superior to Triatix^®^ and it is inferior if the upper bound is below the margin of non-inferiority. The study will be inconclusive if the upper and lower bounds of the confidence intervals are outside the margin of non-inferiority.

### 3.14 Modelling the transmission dynamics of *Theileria parva* and *Anaplasma marginale* in cattle, and the epidemiological impact of Mazao Tickoff

We will use the stochastic susceptible-infectious-recovered (SIR)-based model to understand the transmission dynamics of *T*. *parva* and *A*. *marginale* in cattle, and to model the epidemiological impact of Mazao Tickoff. In this model, the cattle population will be classified as either susceptible (S_c_), infected and infectious (I_c_), or recovered and carrier (C_c_). The tick population will be divided into three subclasses according to their lifecycle stage: larvae, nymph and adult classes, with each stage containing a susceptible (S_T_) and infectious (I_T_) class.

During the study, the numbers of infectious and susceptible cattle in each herd will be observed and recorded at the start of each observation interval (four-time intervals i.e., Days 0, 60, 120 and 182 of the trial). Animals will be registered as a new case (incidence) on the date they are reported with TBD (either anaplasmosis or East Coast fever and confirmed using HRM-PCR and sequencing).

Further, a mathematical model will be developed to simulate the epidemiological and entomological impact of Mazao Tickoff, using the obtained trial data as critical model input.

### 3.15 Ethics approval and consent to participate

#### 3.15.1 Ethics approval

The trial protocol has been approved by Kenya’s Veterinary Medicines Directorate (Approval reference: MOALF/SDL/VMD/TRIALS/VOL1/14), Directorate of Veterinary Services (no objection ref: MOALF/SDL/DVS/DS/RES/74), Pwani University Ethics Review (approval number ERC/EXT/002/2020), National Commission for Science, Technology and Innovation (NACOSTI; License No: NACOSTI/P/21/6726), and ICIPE’s Institutional Animal Care and Use Committee (IACUC, Reference No. Oundo-icipeACUC-Mar2020). Any deviation from the protocol that will impact the conduct of the study will also be immediately reported to VMD and DVS, ICIPE*’s* IACUC, and NACOSTI as appropriate.

#### 3.15.2 Informed consent

Before the implementation of interventions, consultative meetings will be held to explain the objectives, study design, details of products under investigation, implementation procedures and expected outcomes of the trial. This will include the DVS and the various administrative levels from the County level up to the village level. Permission through official letters will be obtained from the various administrative levels. Participating cattle owners will then be required to sign an informed consent form (S1 File) allowing enrolment of their cattle into the study. If the signatory is illiterate, a thumbprint will be obtained and confirmed by an independent witness. All consent forms will be countersigned by the staff member obtaining consent and a copy will be left at the household.

### 3.16 Trial oversight

No data safety and monitoring board will be installed for this trial. Triatix^®^ forms part of the routine tick control program in Kenya and will be undertaken in collaboration with Kenya’s Directorate of Veterinary Services in the locality of the trial. There are no apparent risks to safety for humans, animals, and the environment in the use of Mazao Tickoff, as per the toxicity and eco-toxicity results of the *M*. *anisopliae* isolate ICIPE 7 (https://patents.google.com/patent/WO2017216752A1/en). Kaaya et al. [18] also reported no adverse reactions in cattle treated with fungal formulations of *M*. *anisopliae* at any time during the experiment. It was also observed that all cattle gained significant body weight during the experimental period, suggesting that fungal entomopathogens are safe for cattle. Further, the fungus *M*. *anisopliae* in aqueous or oil formulations is known to pose negligible risk to humans, animals and the environment [43, 57]. Therefore, we do not anticipate any adverse effects on the cattle or humans from the Mazao Tickoff bioacaricide. However, all personnel responsible for spraying the cattle with acaricides will be provided with the appropriate personal protective equipment (PPE).

During the trial, the principal investigator or a delegated person will make regular field visits for quality control of the work done in the field. During these visits, the investigator will monitor all aspects of the trial, including adherence to the standard operating procedures (SOP), documentation, and record-keeping.

Herds could be withdrawn prematurely from the study due to the owner’s decision to withdraw consent; or at the discretion of the investigator for reasons that include non-compliance with the study protocol (for instance, treatment with a study-proscribed acaricidal product), or development of a serious illness that is incompatible with continuation in the study.

## 4. Discussion

The long-term sustainability of tick control programs achieved through the use of chemical acaricides is threatened by the development of acaricide resistance in tick populations [13, 14, 58]. Thus, there is an urgent need to develop alternative and sustainable control methods to manage tick infestations on livestock.

Previous studies have suggested the use of entomopathogenic fungi *M*. *anisopliae* as an alternative tool in tick control and resistance management [18, 25]. However, none of these studies have used a randomized controlled design to provide clear evidence of their efficacy. The new Mazao Tickoff bioacaricide has not been tested using robust trial designs in natural field conditions beyond the pilot trial (https://patents.google.com/patent/WO2017216752A1/en). To this end, this will be the first randomized controlled trial designed to test the efficacy of this novel bioacaricide product against natural tick infestations on cattle. Our results will be discussed as per reference to similar studies that have used same fungal based products or fungal species for field efficacy trials [59–61].

The primary outcome of interest for this trial will be the assessment of the percentage reduction in tick counts in bioacaricide-treated cohorts as compared to placebo at each post-treatment follow-up visit. For this, tick counts will be performed at two-week intervals throughout the trial period. This counting frequency is ideal for three reasons. First, the engorgement period for adult *Rh*. *appendiculatus* is 7–9 days [62] while in adult female *Am*. *variegatum* ticks is 9–12 days [63, 64] with the males remaining on their host for several months [63, 65]. Secondly, the lethal time for 50% mortality (LT_50_) in *M*. anisopliae ICIPE 7 is 2.6 ± 0.3 days in amitraz-susceptible *Rh*. *decoloratus* strain and 3.1 ± 0.5 days in amitraz-resistant strain [25]. Thirdly, our previous pilot trial in the Hargeisa region in Somaliland found that *M*. *anisopliae* ICIPE 7 was viable for a week in the field (http://www.icipe.org/publications/biopesticides-effective-tools-control-ticks), with persistence varying among the different parts of the animals: ribs (63%), spine (66%), udder/scrotum (69%) and ear (72%) 7 days post-treatment. Elsewhere, *M*. *anisopliae* persisted on cattle ears for 3 weeks [20]. Hence, the application of Mazao Tickoff once every two to three weeks could be economical to achieve effective control of ticks on cattle.

Our preliminary cross-sectional survey in the trial area conducted during the dry season found that the median infestation intensity on an animal was six ticks and an average herd size of 7 animals. Considering cattle in trial areas graze communally and obtain water in the same area at respective villages, we will require evidence that these cattle visit tick-infested areas. Hence, we will recruit herds with at least one animal with a live-attached hard tick(s). As such, we will not necessarily consider cattle with tick numbers above the median infestation intensity only.

Previous studies have reported that *Rh*. *appendiculatus*, *Rh*. *evertsi*, *Rh*. *decoloratus* and *Am*. *variegatum* are most abundant on their hosts during the rainy seasons, and soon thereafter than during the dry seasons [3, 38–41]. Thus, in this trial, post-treatment live tick counts on cattle will be done every two weeks for 6 months covering both the dry and rainy seasons. This long period of follow-up with repeated measurements is more likely to give a true picture of the efficacy of the Mazao Tickoff as compared to short follow-up durations covering only one season.

Tick species may differ in susceptibility to entomopathogenic fungi, and, therefore, the efficacy of the Mazao Tickoff may vary according to the tick species. *Metarhizium anisopliae* has demonstrated virulent activity against all stages (egg, larva, nymph and adult) of *Am*. *variegatum*, *Rh*. *appendiculatus*, *Rh*. *evertsi*, *Rh*. *microplus* and *Rh*. *decoloratus* through contact effect [16, 18–21, 66]. Our previous cross-sectional survey in the trial area found that the tick species naturally infesting cattle are *Am*. *gemma*, *Am*. *variegatum*, *Rh*. *appendiculatus*, *Rh*. *evertsi*, *Rh*. *pulchellus*, *Rh*. *microplus*, *Hyalomma rufipes* and *Hy*. *albiparmatum*. In this trial, we will test their susceptibility to *M*. *anisopliae*.

To avoid bias in outcome assessment and consequent data analysis, the field teams that will be implementing the interventions will be separated from those that will be conducting outcome assessment. The outcome assessors will be sending the tick count data using an electronic device pre-installed with RedCap software and will be denied access to the database. They will also not get a preview of the previous tick count. Further, the statistician will be blinded to the intervention arms using intervention arm identification codes.

Analysis of epidemiological outcomes is necessary to demonstrate the efficacy of Mazao Tickoff intervention in protecting the cattle population from contracting tick-borne infections. It may not be possible to evaluate the effects of Mazao Tickoff on the pathogen abundance in ticks after application. This is because it will entail subsampling ticks from the cattle to assess pathogen abundance and this may be a source of bias while assessing tick loads on the cattle, and consequent data analysis. However, we postulate that Mazao Tickoff will result in a reduction in tick burden on cattle and that this, through a reduction in the vector-to-host ratio, will translate into reduced transmission and incidence of tick-borne pathogens. This will be measured using the sequential seroincidence studies (i.e., to assess seroconversion) and the molecular prevalence (active infection) of *T*. *parva* and *A*. *marginale* in cattle. Prevalence will be assessed using surveys at four time-points, i.e., at Days 0, 60, 120 and 182 of the trial. These time-points were deemed appropriate since the incubation period for East Coast fever (*T*. *parva* infection) is eight to twelve days (https://www.galvmed.org/livestock-and-diseases/livestock-diseases/east-coast-fever/) while the incubation time for anaplasmosis (*A*. *marginale*) varies from two weeks to over three months, but averages three to four weeks (https://www.thecattlesite.com/diseaseinfo/255/anaplasmosis/). Once an animal recovers from these infections, either naturally or with normal therapy, it will usually remain a carrier of the disease for life. The two pathogens were chosen because our previous cross-sectional survey in the proposed study area found that they are the most prevalent in cattle (unpublished). By gathering data on multiple outcomes, i.e., pathogen abundance and tick burden, we anticipate that it will be possible to attribute an effect on disease incidence and pathogen prevalence to the Mazao Tickoff intervention.

A non-inferiority study can be used to determine whether a new product is as effective as an existing product at a lower cost or with fewer unwanted side effects. In this trial, we will compare the acaricidal efficacy of Mazao Tickoff with the commonly-used Triatix^®^ acaricide in a non-inferiority test. The null hypothesis for this trial is that the Mazao Tickoff treatment is inferior to the chemical Triatix^®^ treatment by a 10% difference. This margin of non-inferiority is considered acceptable given the limited availability of possible alternatives for tick control and the added advantages of *M*. *anisopliae* as a biological tool for tick control over chemical acaricides. *Metarhizium anisopliae* is selective and virulent to all tick stages [16–22, 42], virulent to acaricide-resistant ticks [25, 67], and is safe to humans, animals and the environment [43]. Further, their compatibility and synergistic potential with various classes of chemical acaricides [25, 60, 67] allows them to be used in the integrated control of livestock ticks. As heavy reliance on chemical acaricides has resulted in health hazards in humans, environmental contamination and contamination of milk and meat products with residues [15], replacement with Mazao Tickoff bioacaricide is likely to mitigate these negative impacts.

During the trial, we will conduct a fungal activity assay every two weeks to assess the virulence and persistence of Mazao Tickoff against the different tick species. This mycosis test will ascertain if indeed our bioacaricide product is the one killing the ticks and that there are no other confounding variables. The fungal activity will be monitored in the laboratory where ticks will be sampled every week and incubated in a controlled environment to allow germination of fungal conidia. If no germination occurs on the cadavers, it will mean that the ticks are possibly dying of other factors and not the bioacaricide product. Further, a mycosis study will help to assess if there is any spillover effect to other intervention arms. For instance, if the fungal activity is detected in the Triatix^®^ or placebo arm, it will indicate that there is cross-contamination among the intervention arms.

This multi-center randomized controlled field trial may create logistical challenges, particularly when herds are required to move for a longer distance to access the crushes where treatments are administered. Therefore, for logistical convenience, we will construct three crushes in each village cluster and all treatments will be administered (per herd) in each crush. We will account for village and herds effects by including herd nested within village as a random effect during statistical analysis. We aim to minimize contamination by allocating one intervention per herd. However, it would be practically impossible to prevent herds of different intervention arms from sharing the common/communal grazing fields, watering points and paths during this trial. Nonetheless, we do not anticipate any influence on the results due to these interactions.

A major obstacle in using entomopathogenic fungi under field conditions is the rapid inactivation or loss of viability of the conidia caused by ultra-violet (UV) radiation, humidity and extreme temperatures [68, 69]. Mazao Tickoff is a broad spectrum acaricidal product specially formulated to improve its efficacy and increase the viability and persistence of the conidia under field conditions. In the bioacaricide formulation, the lower rate of 2.5–3.5% kerosene has a repellent effect on ticks and hence enable the formulated bioproduct to prevent new tick attachments on the cattle (https://patents.google.com/patent/WO2017216752A1/en). Therefore, in addition to the killing effect of Mazao Tickoff on the host, it also prevents new attachments and consequently engorgements since the attached ticks will also drop after application. Triton X-100 is a nonionic surfactant for the formulation that lowers the surface tension between the conidia and the solvent. This bioacaricide also contains canola oil which increases the viability and persistence of the conidia in the environment by protecting them from unfavorable UV radiation and hence increasing the efficacy of the formulation under field conditions. Canola oil also forms a film on the cuticle which retains moisture for longer periods thus creating good conditions for elevated numbers of conidia to invade the vector under sub-optimal humidity. Furthermore, the oil promotes greater adhesion of hydrophobic conidia to the tick’s lipophilic cuticle [24, 70].

One of the limitations of this trial is that livestock farmers will not be masked or blinded to their allocated intervention due to the nature of the intervention products. This may cause non-compliance and even dropouts during the trial, especially in the placebo arm. To minimize the rate of dropouts, and hence loss to follow-up, a considerable effort will be given to continuous dialogue with the community to maintain adherence throughout the study. To minimize non-compliance by livestock farmers, e.g. the use of chemical acaricide in the placebo and Mazao Tickoff treatment arms, all cattle in the herd will be treated with the same product irrespective of their tick infestation status at day 0. Moreover, the project will provide farmers with incentives in the form of strategic deworming (twice during the project period) of their cattle, and veterinary consultancy and free treatment of their cattle if they are found with suspected symptoms of trypanosomiasis or tick-borne diseases during the trial period. The placebo arm (excipients of Mazao Tickoff) will benefit by being given the Mazao Tickoff at the end of the study period.

This study is expected to generate empirical evidence to support the efficacy of Mazao Tickoff bioacaricide as a sustainable tool for tick and tick-borne diseases control on livestock. If proven effective, the next step will be to scale up the usage of Mazao Tickoff to other geographical regions with different climatic factors, tick species, tick burden, cattle breeds and herd management practices.

## Supporting information

S1 File (DOCX)

## Abbreviations

DVS: Directorate of Veterinary Services
EDTA: Ethylenediaminetetraacetic acid
HRM-PCR: High-resolution melting polymerase chain reaction
ICIPE: International Centre of Insect Physiology and Ecology
ITT: intent-to-treat
NACOSTI: National Commission for Science, Technology and Innovation
PP: per-protocol
PPE: personal protective equipment
RCT: Randomized Controlled Trial
REDCap: Research Electronic Data Capture
TBDs: Tick-borne diseases
VMD: Veterinary Medicines Directorate
