# Supplementary material for: Evaluating the efficacy of Mazao Tickoff (Metarhizium anisopliae ICIPE 7) in controlling natural tick infestations on cattle in coastal Kenya: Study protocol for a randomized controlled trial
Source: PLoS One. 2022 Aug 16;17(8):e0272865. doi: 10.1371/journal.pone.0272865 (PMC9380929; doi:10.1371/journal.pone.0272865)
Supplement: S1 File — (DOCX) [file pone.0272865.s001.docx]

**INFORMED CONSENT FOR TICK CONTROL TRIAL IN COASTAL KENYA**

Version Date: 30 July 2021

**Title of the study:** Evaluating the efficacy of Mazao Tickoff (*Metarhizium anisopliae* ICIPE 7) in controlling natural tick infestations on cattle in coastal Kenya.

**Institution**: International Centre of Insect Physiology and Ecology (*icipe*)- Duduville Campus Nairobi.

**Duration of the project:** 16 September 2021 – 16 March 2022

**Principal Investigator:** Daniel Masiga, PhD

*Principal Research Scientist and Head, Animal Health Theme, icipe*

**Co-Investigator:** Shewit Kalayou, DVM, PhD

*Research Scientist, Animal Health Theme, icipe*

*You are being asked to participate in this study by authorizing your cattle to be enrolled in the trial. The purpose of this consent form is to give you the information necessary to help you decide whether or not to be in the study. Please read this form carefully. You are allowed to ask questions about the research or this form that is not clear.*

**Introduction to research**

Ticks are parasitic arthropods that are present in most regions of the world.  They feed on the blood of mammals, birds, and occasionally reptiles and amphibians, with the potential of transmitting diseases such as East Coast Fever, babesiosis, anaplasmosis, cowdriosis and Q fever in livestock.  They also transmit Crimean-Congo hemorrhagic fever, Q fever, tick typhus, African tick-bite fever, etc. in humans. Global losses to livestock due to tick and tick-borne diseases (TBDs) are estimated at US$ 13.9-18.7 billion annually, and over 800 million cattle worldwide are constantly exposed. Direct losses caused by heavy tick infestation on cattle include damage to hides and skins, damage to teats and reduced productivity, decrease in live-weight gain, blood loss, suppression of immunity, tick worry and introduction of toxins.

The use of chemical acaricides remains the primary approach to the management of TBDs. However, ticks have developed resistance to most of the major products, in addition to concern over the effects on the environment. Real IPM and *icipe* are developing a fungus-based acaricide (Mazao Tickoff) as an alternative to chemical acaricides and as a tool for resistance management. This fungus-based acaricide affects insects only. It has no safety risk to humans, animals and the environment.

If we find that Mazao Tickoff is able to decrease natural tick infestation levels on cattle, then this could be used as an alternate or complementary method for tick control in this region and throughout Kenya and beyond.

**Study procedures**

In this study, we will allocate your cattle to receive either Mazao Tickoff, amitraz (Triatix®), or placebo (excipients of the Mazao Tickoff®). These will be administered topically to your cattle at a two-week interval for 6 months. Further, there will be whole-body tick counts on the animals every two weeks for 6 months. Additionally, the animals will be examined during the entire duration of the trial to document any adverse events that could be interpreted as an adverse reaction to bioacaricide treatment.

**NB**: You will not be allowed to use any non-study products during the trial period.

**Risk of the study**

There are no apparent risks to the safety of human, animal and the environment in the use of Mazao Tickoff, as per the toxicity and eco-toxicity results of the *M. anisopliae* isolate ICIPE 7 (<https://patents.google.com/patent/WO2017216752A1/en>).

**Benefits/ compensation of the study**

Where we suspect your animals are infected by any tick-borne pathogen, we will provide diagnosis and treatments through administration of veterinary drugs. You will not be charged for this or any other procedures performed solely for study purposes. Any other unrelated diagnosis treatment and management are your responsibilities.

**Voluntary participation and withdrawal**

You have the right to decide whether you want to participate or not. If you choose to participate in the study, we will ask you to sign this consent form. You may withdraw your participation in the study at any time without any penalty.

**Confidentiality:**

Your personal information and that of your animal will be kept confidential. Your identity and those of your animals will not be revealed when reporting or publishing the data arising from the study. The consent forms will be kept in a locked cabinet that will only be accessed by authorized personnel. Samples will be given a unique identity which is only understood by the laboratory staff.

**Contact persons**

If you have any further questions about this research, you can contact the principal investigator Dr. Daniel Masiga (email: dmasiga@icipe.org). This project has been approved by the Veterinary Medicines Directorate (VMD) and Directorate of Veterinary Services (DVS). If you have concerns about your rights as a research participant, or concerns or complaints about the research, you may contact:

Veterinary Medicine Directorate,

P.O Box 66171-00800 Westlands-Nairobi,

Tel: +254743795395,

Email: [vmd@kilimo.go.ke](mailto:vmd@kilimo.go.ke)

**OR**

Directorate of Veterinary Services,

State Department of Livestock,

Ministry of Agriculture, Livestock and Fisheries

P.O Private Bag, Kangemi 00625 Nairobi, Kenya

Tel: 020 – 8043441

Email: [infodvs@kilimo.go.ke](mailto:infodvs@kilimo.go.ke)

**TICK CONTROL TRIAL**

**WRITTEN ASSENT FORM**

Version date: 30 July 2021

I, , was invited to participate in the research to evaluate

(Name)

the efficacy of Mazao Tickoff in controlling natural tick infestations on cattle in coastal Kenya.

I have heard and read the information sheet and the consent form in the presence of an independent witness. I have also received an adequate explanation of the purpose/methodology of the study, possible risks and benefits that may occur to me upon participation in the study, to my satisfaction and understanding. I have accepted to have my animals take part in this trial freely and I can also choose to withdraw out of the study at any time.

I have read and understood all statements in this consent form before signing my name.

Name Signature Date

Name of Investigator Signature Date

Thumb mark for illiterate participants

Witness testimony:

The objective, procedures, risks and benefits of the study have been explained to the participant and he/she was encouraged to ask questions for clarifications. The participant was also advised that he/she may not participate in the research study and may withdraw their participation at any time. This would not affect him/her or their family. The participant was also informed that his/her identification shall be kept confidential and shall be known only to the researchers.

Name of Witness Signature Date
